# Evaluation of bottom-up interventions targeting community-dwelling frail older people in Belgium: methodological challenges and lessons for future comparative effectiveness studies

**DOI:** 10.1186/s12913-019-4240-9

**Published:** 2019-06-24

**Authors:** Anne-Sophie Lambert, Sophie Ces, Espoir Bwenge Malembaka, Thérèse Van Durme, Anja Declercq, Jean Macq

**Affiliations:** 1 Institute of Health and Society (IRSS), Université Catholique de Louvain, Clos chapelle aux champs 30 /B1.30.15.05, 1200 Brussels, Belgium; 2 Ecole Régionale de Santé Publique (ERSP), Faculty of Medicine, Université Catholique de Bukavu, Bukavu, Democratic Republic of Congo; 3 LUCAS and Center for Sociological Research, KU Leuven, Leuven, Belgium

**Keywords:** Comparative effectiveness studies, Bottom-up interventions, Stratification of population, Aging, Belgium

## Abstract

**Background:**

Optimizing the organization of care for community-dwelling frail older people is an important issue in many Western countries. In Belgium, a series of complex, innovative, bottom-up interventions was recently designed and implemented to help frail older people live at home longer. As the effectiveness of these interventions may vary between different population groups according to their long-term care needs, they must be evaluated by comparison with a control group that has similar needs.

**Methods:**

The goal was to identify target groups for these interventions and to establish control groups with similar needs and to explore, per group, the extent to which the utilization of long-term care is matched to needs. We merged two databases: a clinical prospective database and the routine administrative database for healthcare reimbursements. Through Principal Component Analysis followed by Clustering, the intervention group was first stratified into disability profiles. Per profile, comparable control groups for clinical variables were established, based on propensity scores. Using chi-squared tests and logistic regression analysis, long-term care utilization at baseline was then compared per profile and group studied.

**Results:**

Stratification highlighted five disability profiles: people with low-level limitations; people with limitations in instrumental activities of daily life and low-level of cognitive impairment; people with functional limitations; people with functional and cognitive impairments; and people with functional, cognitive, and behavioral problems. These profiles made it possible to identify long-term care needs. For instance, at baseline, those who needed more assistance with hygiene tasks also received more personal nursing care (*P* < 0.05). However, there were some important discrepancies between the need for long-term care and its utilization: while 21% of patients who were totally dependent for hygiene tasks received no personal nursing care, personal nursing care was received by 33% of patients who could perform hygiene tasks.

**Conclusions:**

The disability profiles provide information on long-term care needs but not on the extent to which those needs are met. To assess the effectiveness of interventions, controls at baseline should have similar disability profiles and comparable long-term care utilization. To allow for large comparative effectiveness studies, these dimensions should ideally be available in routine databases.

**Electronic supplementary material:**

The online version of this article (10.1186/s12913-019-4240-9) contains supplementary material, which is available to authorized users.

## Background

The health status of older people is often characterized by the interplay between frailty, multi-morbidity, and disability. Frail older people are usually more vulnerable to stressors as a consequence of a significant reduction of physiological reserves [1, 2]. Multi-morbidity is defined by the presence of two or more chronic conditions [3]. The World Health Organization (WHO) defines disability as “an umbrella term for impairments, activity limitations or participation restrictions” [4]; the last-named refers to difficulties in carrying out essential tasks for independent living and is assessed by the activities of daily life (ADL) and instrumental activities of daily life (IADL) scales [5]. However, it is worth noting that disability is strongly influenced by cognitive status [6, 7].

These three distinct concepts are strong determinants of service needs and utilization, and more specifically of home care support [5]. Frailty is associated with a higher utilization of both health services–including the primary care providers, clinical specialist physicians, hospital admissions, and emergency department visits–and home care services such as nursing care, home help, and meals-on-wheels [8]. People with multi-morbidity consult specialists and general practitioners more often and are more frequently admitted to hospital, where they have longer stays, on average, than people without multi-morbidity [3]. Multi-morbidity is also associated with long-term care needs. Indeed, long-term care needs are high in people suffering from neurological disorders such as dementia and Parkinson’s disease, as well as in people who have had a stroke or suffer from diabetes or oncological conditions [9]. However, the level of disability is the foremost determinant of long-term care expenditure. The level of disability has different repercussions on long-term care needs [10] and determines the necessity to start using home care and to move from home care to a nursing home [11].

Finally, health services utilization is uneven among people with similar needs. Two situations of mismatch between needs and services utilization may be at play. First, the overuse of healthcare services that are unlikely to improve or may even negatively affect people’s health, what are called “lower-value services” [12]; and second, the underuse of health and social services that would improve people’s health. As a consequence, patterns of health service utilization should be analyzed in the light of service (mis)use.

In Belgium, the number of older persons and the number of frail or dependent older persons with multi-morbidity will rise in the coming years [13]. An increasing number of dependent people are expected to remain at home, because of the expected shortage of beds in nursing homes [14]. The majority of older people also prefer to remain in their own surroundings, rather than be admitted to a nursing home [14]. Consequently, older people with many more disabilities will live at home, supported by both formal and informal caregivers. Against this background, a nationwide program (Protocol 3 (P3)), which consists of a set of innovative, bottom-up-designed interventions that include case management, occupational therapy at home, psychological support, and respite care, were financed by the National Institute of Health and Disability Insurance and were implemented in the form of pilot projects [15]. Case management intervention consisted of (a) an individualized assessment of needs and preferences, (b) planning of services, according to the results of this assessment, (c) patient-centered care coordination, and (d) re-evaluation and adjustment of care coordination. Occupational therapy provided mainly home adaptation in order to: (1) adapt patients’ living environments to their current conditions and (2) offer better work conditions to home caregivers. Psychological support interventions delivered psychological, psychosocial support, and psychotherapy at home, provided by a trained psychologist or psychotherapist. The focus of these heterogeneous interventions was on the support of frail older people at home with the aim of preventing the risk of institutionalization in a nursing home while maintaining a satisfactory quality of life, without increasing the burden on family carers, and fostering a more efficient use of health and social care services [15].

The evaluation of these interventions consisted of describing them, identifying the facilitators and barriers to their implementation, and assessing their impact on clinical outcomes, service utilization, and cost for different sub-populations. Given the heterogeneity of both the interventions and the targeted populations, a comparative effectiveness design and multi-embedded case studies were considered to be more appropriate than a randomized control trial. This paper discusses the methodological challenges of the comparative effectiveness study; the case studies are the focus of another paper [16]. We used an observational longitudinal study design, with routine data and prospective data collection, to compare frail older people receiving care and benefiting from different types of interventions (intervention group) with a group of study participants benefitting from “routine care” (control group).

Two challenges are usually encountered in designing comparative effectiveness studies: first, the relevant stratification of the overall sample so as to identify the most suitable intervention per sub-group of frail older people; second, the definition of an adequate control group in order to evaluate the consequences of the interventions.

The identification of the long-term care needs of frail older people should help in identifying appropriate variables to use in defining population subgroups and creating a control group with characteristics similar to those of the intervention groups. As mentioned above, this can be done by looking at disability, family caregiving, and health services utilization. This paper will therefore propose a systematic methodological approach to addressing the following questions:

- Is it possible to identify the need for long-term care? Can this lead to a meaningful stratification of frail older people and a clear definition of a control group?
- To what extent does the utilization of long-term care match the need for long-term care?
- Is it possible to use a routine database of healthcare reimbursements to select controls with long-term care needs similar to those of the intervention groups?

## Methods

### Overall methodological approach

We developed a step-by-step methodological process combining different approaches. First, we divided the intervention group into intragroup-homogeneous strata to define different disability profiles. Second, we carried out a one-by-one matching of the participants from the intervention group with the control group to account for people with similar long-term care needs. Third, we compared healthcare utilization between the groups studied in order to evaluate the baseline difference in how widely those needs are met (low and high value).

### Inclusion criteria

The inclusion criteria for selecting participants in the intervention and the control groups included: [17] being at least 60 years old and scoring at least six on the Edmonton Frail Scale [18]; having a dependency status of A, B, or C on the Katz home scale or a B, C, or Cd status on the Katz residential scale [19]; or having been diagnosed with dementia by a geriatrician, neurologist, or psychiatrist. People in both groups were living at home at baseline.

### Characteristics of the databases used

Two databases were available and were merged with national registration numbers for this study.

The first is the BelRAI database which is the Belgian version of an internationally validated geriatric comprehensive assessment, the interRAI Instrument Home Care version (interRAI HC) [20]. This provided prospective data on the main clinical variables. Functional performance in activities of daily life (ADL) was evaluated by four items (personal hygiene, toilet use, locomotion, and eating) with a total score varying from 0 to 6. A total score above 3 (cut-off of ADL scale) indicates major dependency; 6 is the maximum level of dependency [21]. Functional performance in instrumental activities of daily life (IADL) was evaluated by eight items (meal preparation, ordinary housework, managing finances, managing medications, phone use, stairs, shopping, and transportation) with item scores varying from 0 to 6 and total scores varying from 0 to 48 [22]. A total score above 24 (cut-off of IADL scale) indicates major dependency; 48 is the maximum level of dependency. Cognitive status was evaluated by a cognitive performance score (CPS), which consisted of four items (decision-making, short-term memory, procedural memory, and comprehension). The total score ranges from 0 to 6; a total score above 3 (cut-off of CPS scale) indicates major cognitive impairment [23]. The depression rating scale (DRS), with total scores ranging from 0 to 14, was used to evaluate depression symptoms. A total score above 3 (cut-off of DRS scale) indicates significant depressive symptoms; a score of 14 indicates the presence of all mood symptoms in the last 3 days [24]. Behavioral problems (Behav.) were evaluated by the Aggressive Behavior Scale (ABS), which includes five items (wandering, verbal aggression, physical violence, abnormal social behavior, and resistance of care) [25]. The total score varies from 0 to 12 [25]. A total score above 0 indicates behavioral problems (cut-off of Aggressive Behavior Scale); a score of 12 indicates frequent and varied behavioral problems [25]. The level of presence of a family carer, the Zarit Burden interview, and the WHO-QoL-8 were added to interRAI Instrument in the BelRAI database. The level of presence of a family carer was encoded, according to living arrangements, as a categorical variable with three levels (without a family carer, with a non- resident family carer, and with a co-resident family carer). This categorization allows the differentiation of the intensity of informal caregiving provided [26]. The 12-item version of the Zarit Burden interview (ZBI-12) was used to estimate the burden on family carers. This scale contains 12 items, with scores from 0 to 4. The total score ranges from 0 to 48; higher scores are indicative of greater burdens on family carers. A total score above 10 (cut-off of ZBI-12 scale) indicates that a family carer has significant depressive symptoms [27]. The WHO- QoL-8 was used as a concise instrument to measure the client’s perceived generic quality of life [28]. Finally, an ad-hoc economic questionnaire was used to measure social care services utilization and informal aid. The interRAI HC instrument was filled out at enrolment, at the exit of the patient from the project, and 6 months after enrolment [15]. Only the baseline evaluation data were used for this paper.

Secondly, the IMA database from the Inter-mutuality Agency provided routine data on the healthcare services reimbursed by the National Insurance for Health and Disability Institute (NIHDI).

These two merged databases were cleaned to contain only complete evaluations at baseline. Data were available for 10,783 beneficiaries of Protocol 3 intervention (intervention group) and 605 older persons benefitting from “usual care”, who were recruited at home by nursing care organisations (control group). Of the beneficiaries of Protocol 3 intervention, 1811 lived at home and had no family carer, 5735 had a non-resident family carer, and 3842 had a co-resident family carer. In the control group, 78 lived at home and had no family carer, 268 had a non-resident family carer, and 259 had a co-resident family carer.

In addition, the median fiscal income per household by municipality (data from statbel.fgov.be) was used as a proxy for socioeconomic level. Belgian municipalities were classified in three categories by using the first and the third quartiles of the median fiscal income per household. Municipalities included in the first quartile, accordingly, have a low median fiscal income per household. The municipalities included between the first and the third quartile, have a medium median fiscal income per household. And the municipalities in the last quartile have a high median fiscal income per household.

### Stratification of the intervention group

In order to identify population groups with specific long-term care needs, we identified “natural” clustering of individuals with a similar disability profile in the intervention group, using statistical analysis to establish classification schemes [29].

The variables used for the establishment of classification schemes were inspired by existing classifications in which clinical scales (ADL, IADL, depression, and cognition) and comorbidity were included [30–32]. These classifications highlighted the fact that cognitive and functional limitations fit the different disability profiles better than comorbidities [30]. Indeed, the need for long-term care services is associated with specific limitations [10] (i.e. significant IADL limitations are associated with needing household aid services (domestic help, home care worker, or meals-on wheels, etc.); significant ADL limitations are associated with needing personal nursing care; and significant cognitive impairment is associated with needing supervision [33]). Hence, the comorbidities were not included in our classification. A specific scale for behavioral problems was included in our classification because people with behavioral problems represent a population with specific health and social care needs, including the need for supervision by family carers and the need for respite care for those carers [34]; in addition, this population is likely to make less use of support services [34] and imposes a greater burden on family carers [35].

The “natural” clustering was, consequently, established by combining the different scores on functional limitations, cognitive performance, and the presence of behavioral problems in a Principal Component Analysis (PCA), followed by a cluster analysis (based on the PCA correlation matrix between the initial variables and the principal components) [36]. A hierarchical algorithm (Ward algorithm) was used to define the number of groups by the decomposition of the inertia of the cloud of points while minimizing the loss of information at each additional clustering. The number of groups obtained was the result of a trade-off between intra-group homogeneity and inter-group heterogeneity [36]. This method was employed using the FactoMineR package [37] in R. Disability profiles were presented by means of the descriptive statistics of the BelRAI scales. For each scale, the median and interquartile range, both for the total score and for each item, and the proportion of individuals above the total score cut-off were presented.

### One-by-one matching of participants from the intervention group with the control group

The control group was constituted by one-by-one matching of P3 beneficiaries with individuals from the control group, based on a similar level of presence of a family carer and maximum similarity in scores on the clinical scales, age, and sex.

The establishment of the control group followed a multi-stage process. The initial step consisted of two successive stratifications. First, intervention and control groups were stratified by the level of presence of a family carer to ensure similarity of the groups studied with regard to this variable. The rationale behind this initial step was twofold. First, older people with co-resident family carers were frailer than both those without family carers and those with non-resident family carers [38]. Second, the level of utilization of health and social services is associated with the level of presence of a family carer [39]. Within the intervention group, each group of participants with a particular level of presence of a family carer was, in turn, stratified by disability profile. Then a model of propensity scores (with binary dependent variable intervention vs control groups) was created for each stratum, following a step-by-step approach. Each step consisted in a forward selection of a variable among those obtained from the BelRAI scales (ADL, IADL, CPS, DRS, presence of behavioral problems) and the predisposing factors. A likelihood ratio test was then performed to determine whether there was a significant difference between the intervention and control groups with regard to the variable added. A new variable that significantly improved the model was retained. The matching function used was the one-to-one matching of the nearest neighbors with replacement to make it easier to find the best match for a P3 beneficiary among all the potential controls. This was mandatory in our case because of the smaller size of the control group relative to the intervention group. The Matching package in R was used for this purpose [40].

Finally, the evaluation of the covariate balance was carried out using the standardized mean difference (smd) and the analogous variance ratio (VR). To evaluate multiple covariates balance, the average standardized mean difference (SMD) and the Geometric Mean Variance Ratio (GMVR) were used [41, 42].

### Relationship between disability profiles and healthcare utilization

#### Identification and selection of relevant IMA-AIM proxies of chronic disability profiles

The selection of proxies of disability was done by experts (geriatrician, liaison nurse, clinical pharmacist) based on two questions: (1) which kinds of healthcare utilization can be identified as signs of a long-term disability in older people? (2) Which kinds of healthcare utilization allow us to distinguish between different disability profiles among older people?

To identify chronic disability status, proxies were defined based on a one-year observation period before inclusion in a P3 project. By way of illustration, medication was considered chronic when a medicine was taken for more than 3 months in a given year. This selection was confirmed by a univariate test assessing whether proxies defined as binary variables were associated with the risk of institutionalization. The latter was strongly associated with cognitive and functional impairment [43]. Therefore, the factors associated with the risk of institutionalization were considered as good proxies for disability status.

Besides the proxies for chronic disability status, multi-morbidity is also frequently discussed in the literature [5]. The multi-morbidity variables were created from prescription drug data using the Anatomical Therapeutic Chemical (ATC) classification system [44], which recognizes 22 chronic conditions. The presence of two or more chronic conditions in the same individual indicates multi-morbidity [5].

#### Comparison of healthcare utilization between different disability profiles within the intervention group

Proportions of healthcare users were presented to describe the healthcare utilization for the different disability profiles and were compared by a chi-squared test.

#### Comparison of healthcare utilization by groups studied

The difference in proportion of healthcare users between the intervention and the control groups was calculated by a logistic regression for all the proxies, with the intervention group status equaling one and the control group status equaling zero. The odds ratios with their normal bootstrap percentile confidence intervals were adjusted to socioeconomic variables (age, gender, median fiscal income by municipality, and region) and were computed using the Boot package in R [38, 45]. The bootstrapping method used to build the confidence intervals withstands the assumption of normality and equality of variances.

## Results

### Description of the disability profiles of the intervention group

The cluster analysis highlighted five disability profiles (Table 1). The first disability profile (*N* = 2040) was called “low-level limitations (low limit.)”, and consisted of individuals with no significant functional limitation or cognitive impairment.

**Table 1:** Description of the total and item-specific scores of the BelRAI scales for disability profiles

	Max	Low limit.	IADL (cogn.)	Func.	Func., cogn.	Func., cogn., behav.
Activities of Daily Life Scale (ADL)						
% above the cut-off	100	2.99	0.21	70.92	88.86	72.11
ADL score	6	0 [0–0]	0 [0–1]	3 [2–4]	3 [3–4]	3 [2–4]
Personal hygiene	6	0 [0–1]	1 [0–2]	4 [3–5]	5 [4–6]	5 [3–6]
Locomotion	6	0 [0–0]	0 [0–0]	2 [0–4]	3 [0–5]	1 [0–3]
Toilet use	6	0 [0–0]	0 [0–0]	0 [0–3]	3 [0–5]	2 [0–4]
Eating	6	0 [0–0]	0 [0–0]	1 [0–1]	1 [0–3]	1 [0–3]
Instrumental Activities of Daily Life Scale (IADL)						
% above the cut-off	100	4.85	88.61	92.49	99.74	97.03
IADL score	48	14 [8–18]	31 [27–36]	34 [30–39]	44 [40–47]	42 [38–46]
Meal preparation	6	0 [0–3]	5 [3–6]	6 [4–6]	6 [6–6]	6 [5–6]
ordinary housework	6	3 [0–4]	5 [4–6]	6 [5–6]	6 [6–6]	6 [5–6]
Managing finances	6	0 [0–3]	5 [4–6]	5 [2–6]	6 [6–6]	6 [6–6]
Managing medications	6	0 [0–1]	3 [1–6]	3 [0–5]	6 [5–6]	6 [5–6]
Phone use	6	0 [0–0]	0 [0–1]	0 [0–1]	4 [1–6]	5 [1–6]
Stairs	6	0 [0–0]	0 [0–3]	5 [2–6]	5 [2–6]	2 [0–5]
Shopping	6	2 [0–4]	5 [4–6]	6 [5–6]	6 [6–6]	6 [6–6]
Transportation	6	0 [0–4]	6 [4–6]	6 [6–6]	6 [6–6]	6 [6–6]
Cognitive Performance Scale (CPS)						
% above the cut-off	100	5.25	28.42	0.21	82.47	86.8
CPS score	6	0 [0–1]	2 [0–3]	0 [0–1]	3 [3–5]	4 [3–5]
Decision making	5	0 [0–0]	1 [0–2]	0 [0–0]	2 [2–4]	3 [2–4]
Short-term memory	1	0 [0–0]	0 [0–1]	0 [0–0]	1 [0–1]	1 [1–1]
Procedural memory	1	0 [0–0]	0 [0–1]	0 [0–0]	1 [0–1]	1 [1–1]
Understood	4	0 [0–0]	0 [0–1]	0 [0–0]	1 [1–2]	2 [1–3]
Aggressive Behavior Scale (ABS)						
% above the cut-off	100	6.13	13.56	3.51	22.8	100
ABS score	15	0 [0–0]	0 [0–0]	0 [0–0]	0 [0–0]	4 [3–6]
Wandering	4	0 [0–0]	0 [0–1]	0 [0–0]	1 [1–2]	2 [1–3]
Verbal abuse	3	0 [0–0]	0 [0–0]	0 [0–0]	0 [0–0]	3 [0–3]
Physical abuse	3	0 [0–0]	0 [0–0]	0 [0–0]	0 [0–0]	1 [0–2]
Social disruptive behav.	3	0 [0–0]	0 [0–0]	0 [0–0]	0 [0–0]	0 [0–1]
Resistance of care	3	0 [0–0]	0 [0–0]	0 [0–0]	0 [0–0]	0 [0–2]
Depression Rating Scale (DRS)						
% above the cut-off	100	30.25	31.73	19.89	37.26	58.16
DRS score	14	1 [0–3]	1 [0–3]	0 [0–2]	2 [0–4]	3 [1–6]
N						
N	10,783	2040	1932	3821	2316	674

*Max* The total or the items maximum score of the BelRAI scales, *Low limit.* Low-level limitations, *IADL (cogn.)* IADL and low level of cognitive impairment, *Func* Functional limitations, *Func., cogn.* Functional & cognitive impairments, *Func., cogn., behav.* Functional, cognitive & behavioural problems; % above the cut-off is the proportion of individuals above the cut-off of the total score of BelRAI scales; the other lines present the median with the interquartile range of the total or of the items scores of the BelRAI scales

The second disability profile (*N* = 1932) was the “IADL and low level of cognitive impairment (IADL (cogn.)” group. Basically, it consisted of individuals with IADL difficulties, of whom 88% were above the IADL cut-off, with difficulties mainly in preparing meals, performing ordinary housework, managing finances, managing medications, shopping, and transportation. In addition, a majority (55%) of individuals in this group had minor cognitive impairments (CPS score higher than 1), 14% had behavioral problems (mainly wandering), and 32% suffered from depressive symptoms.

The third disability profile (*N* = 3821), referred to as “functional limitations (func.)”, was made up of individuals whose limitations were mostly functional ones. Of these, 92.5% had an IADL score above the cut-off of dependency and 71% had difficulties performing ADL, especially in hygiene tasks. Indeed, up to 64% of individuals in this group needed some help to accomplish 50% of personal hygiene-related tasks.

The fourth disability profile (*N* = 2316) was called “functional and cognitive impairments (func., cogn.)” and consisted of individuals with both functional limitations and cognitive impairment. Up to 100 and 89% of individuals in this group were above the IADL and ADL cut-offs respectively and 82.5% had CPS scores above the cut-off that defines cognitive impairment. Additionally, over 75% of participants in this category were completely dependent for almost all IADL tasks. The main ADL limitation was related to hygiene tasks, with which 86% of the participants needed extensive assistance. Such difficulties were more pronounced in this group than among those grouped under the “functional limitations” disability profile, probably due to the concurrence of functional limitations with cognitive impairment.

Finally, the fifth disability profile (*N* = 674), which was named “functional, cognitive, and behavioral problems (func., cogn., behav.)”, consisted of individuals who combined functional limitations, cognitive impairment, and behavioral problems. In this group, 97% of study participants were above the IADL cut-off, 72% above the ADL cut-off, and 87% above the CPS cut-off. Half of them had behavioral problems, including wandering and verbal abuse on 2 days out of the three prior to the evaluation. Individuals in this group were similar to those grouped under the “functional and cognitive impairments” disability profile with regard to functional aspects, but they suffered from more serious cognitive problems (mainly in decision-making and comprehension).

### Evaluation of the matching balance

The matching balance was respected: all the average standardized mean difference were smaller than 0.25 and all the geometric mean variance ratio were smaller than 2, excepted for strata with a small number of Protocol 3 beneficiaries and a small number of potential controls (e.g., where there were only 29 beneficiaries and one control without informal caregivers and with functional, cognitive, and behavioral problems, or where there were 121 beneficiaries and 9 controls without an informal caregiver and with functional and cognitive impairment) (Table 2). That can be explained by the limited number of people living alone at home who had significant cognitive impairment.

**Table 2:** Evaluation of balance of the BelRAI covariables between study arms by disability profile

Family carer	Low limit.			IADL (cogn.)			Func.			Func., cogn.			Func., cogn., behav.		
	Without	Non-resident	Co-resident	Without	Non-resident	Co-resident	Without	Non-resident	Co-resident	Without	Non-resident	Co-resident	Without	Non-resident	Co-resident
Activities of Daily Life Scale (ADL)															
smd	0.06	0.53	0.32	0.09	0.01	0.2	0.18	0.2	0.14	0.35	0.16	0.15	1.72	0.35	0.34
VR	1.11	1.73	1.95	1.88	1.64	1.03	1.03	1.73	2.26	2.67	1.15	1.43	–	1.68	1.37
Instrumental Activities of Daily Life Scale (IADL)															
smd	0.11	0.36	0.19	0.3	0.05	0.29	0.21	0.11	0.39	0.36	0.01	0.05	1.26	0.35	0.51
VR	1.55	1.1	1.34	1.88	2.18	4.67	1.57	1.69	2.6	1.04	1	1.13	–	1	1.61
Cognitive Performance Scale (CPS)															
smd	0.08	0.1	0.24	0.26	0	0.13	0.39	0.01	0	0.49	0.05	0.12	0.8	0.14	0.74
VR	1.11	1.16	1.03	1.84	1.47	1.59	3.96	1.2	1.01	1.5	1.07	1.18	–	2.21	2.01
Depression Rating Scale (DRS)															
smd	0.57	0.25	0.25	0.27	0.16	0.2	0.03	0.02	0.04	0.44	0.2	0.1	1.44	0.14	0.08
VR	3.18	1.26	2.12	1.26	1.13	1.69	2.01	1.35	1.15	5.86	1.24	1.43	–	1.8	1.12
Aggressive Behavior Scale (ABS)															
smd	0.32	0.2	0.18	0.32	0.12	0.06	0.36	0.16	0.02	0.6	0.26	0.24	0.66	0.13	0.01
VR	–	5.25	1.32	2.36	2.56	1.23	11.91	2.33	1.19	6.47	3.64	2.72	–	1.29	1.26
Average Standardized Mean Difference (SMD)	0.3	0.24	0.24	0.24	0.11	0.21	0.2	0.11	0.12	0.44	0.13	0.15	1.02	0.26	0.32
Geometric Mean Variance Ratio (GMVR)	1.44	1.65	1.56	1.74	1.66	1.6	2.52	1.5	1.42	2.87	1.44	1.42	–	1.54	1.38
Treated															
N	740	965	335	267	1135	530	576	2234	1011	121	919	1276	29	214	431
Control															
N	41	52	27	30	70	49	38	210	147	9	54	144	1	10	24

The evaluation of the balance of the BelRAI covariables was carried out for each of the propensity score models, that is, for each level of presence of an informal caregiver (without a family carer, with a non-resident family carer, with a co-resident family carer) within different disability profiles (*Low limit.* Low-level limitations, *IADL (cogn.)* IADL and low level of cognitive impairment, *Func* Functional limitations, *Func., cogn.* Functional & cognitive impairments, *func., cogn., behav.* Functional, cognitive & behavioural problems). *smd* Standardised mean difference, *VR* Variance ratio, *SMD* Average standardized mean difference, *GMVR* Geometric mean variance ratio, *N* Indicates the number of participants per group studied

### Relationship between disability profile and healthcare utilization

#### Description of healthcare utilization by disability profile in the intervention group

The five disability profiles allow us to identify long-term care needs. However, it was not always clear to what extent the need for healthcare of those with particular disability profiles matched utilization of that care.

People who had used nursing services for hygiene tasks (personal nursing care) more than twice a week for more than 3 months in the year preceding inclusion in the study were considered to be chronic users of nursing care for dependency reasons. The greater the need of assistance with hygiene tasks, the more personal nursing care was used. Personal nursing care was given to 33% of people without hygiene task difficulties, to 52% of those in need of occasional assistance, to 71% of those whose need of assistance was judged to be major or maximal, and to 79% of people with total dependency for hygiene tasks (Table 3). People who used physical therapy for dependency reasons had a prescription as part of the treatment of severe pathologies or for 60 physical therapy sessions after experiencing a fall. This need for physical therapy increased significantly with the need for assistance with locomotion: it was seen in 40% of people needing assistance with locomotion as against 18% of those not in need of assistance with locomotion (Table 3). Parkinson’s medication intake varied significantly between disability profiles, ranging from 5% for individuals with a low-level limitations profile up to 12% for people with high levels of functional and cognitive impairment (Table 3). The same variation was seen in those receiving the incontinence lump sum (a fixed payment system for incontinence material [46]), with proportions ranging from 1% among individuals with low-level limitations up to 23% for people with high levels of functional and cognitive impairment (Table 3).

**Table 3:** Functional IMA proxies by disability profile and level of limitation for specific ADL tasks

	Low limit.	IADL (cogn.)	Func.	Func., cogn.	Func., cogn., behav.	*P* value	Total
Nursing							
Hygiene tasks < 3	25.38	39.52	45.95	38.2	37.21		33.33
Hygiene tasks = 3	48.74	54.01	49.01	65.18	62.96		52.22
Hygiene tasks]3–6]	75.51	100	69.78	73.34	70.83		71.19
Hygiene tasks = 6	42.86	100	77.43	80.67	76.71		78.88
*P* value	–	–	***	***	***		***
Total	29.28	41.65	63.58	74.13	65.51	***	55.54
Physical therapy							
Locomotion *≤*3	9.83	14.99	22.48	24.89	16.76		17.79
Locomotion > 3	0	0	34.45	45.48	41.03		39.83
*P* value	–	***	***	***	***		***
Total	9.81	14.99	25.59	33.32	22.32	***	22.02
Parkinson’s medication							
Total	5.45	7.67	8.94	12.03	10.49	***	8.82
Incontinence lump sum							
Total	1.34	2.73	12.1	22.98	16.24	***	10.99

Table presents for each disability profile (*Low limit.* Low-level limitations, *IADL (cogn.)* IADL and low level of cognitive impairment, *Func* Functional limitations, *Func., cogn.* Functional & cognitive impairments, *func., cogn., behav.* Functional, cognitive & behavioural problems) the proportions of people utilizing the service. The p values in columns are obtained from the chi-squared test comparing the disability profiles. The *p* values in lines derive from the chi-squared test comparing the different levels of limitation for specific ADL tasks. ***: *p* value ≤0.001; *NS* No significant; −: minimum expected value < 5

Antidepressant intake was reported in 42% of the study participants (Table 4). It was more frequent (53%) among people whose depression rating score was above the cut-off. However, 38% of people who scored lower on the DRS scale took antidepressants (Table 4). Neuroleptic intake was significant among people with cognitive impairments (31% of those with CPS above the cut-off) and was higher among those with cognitive and behavioral problems, being prevalent in 41% of people who had the “functional, cognitive, and behavioral problems” disability profile. However, their use was not limited to people who were cognitively impaired: 15.5% of those without cognitive problems also took neuroleptics (Table 4). Visits to a neuropsychiatrist (at least one visit in the year preceding inclusion in the study) were more frequent among individuals suffering from cognitive problems than among people with depressive symptoms. On the other hand, consultation of a psychiatrist (at least one consultation in the year preceding inclusion) was more common in people with depressive symptoms than in those who were cognitively impaired. Day care center use was less frequent (3% of the total sample), but was higher in people with cognitive impairments (7%) (Table 4).

**Table 4:** Cognitive and mood IMA proxies by disability profile and level of DRS and/or CPS impairments

	Low limit.	IADL (cogn.)	Func.	Func., cogn.	Func., cogn., behav.	*P* value	Total
Antidepressant							
DRS < 3	33.59	36.99	37.23	43.54	42.61		37.92
DRS *≥* 3	55.02	56.82	50.98	51.49	50.13		52.88
*P* value	***	***	***	***	NS		***
Total	40.07	43.29	39.97	46.51	46.98	***	42.43
Neuroleptic							
CPS < 3	13.68	18.52	14.48	21.50	25.00		15.51
CPS *≥* 3	20.18	23.95	10.00	30.04	43.71		31.11
*P* value	NS	**	NS	***	**		***
Total	14.02	20.09	14.47	28.55	41.24	***	20.12
Neuropsychiatrist							
DRS < 3, CPS < 3	15.20	21.71	19.11	22.14	37.93		18.85
DRS < 3, CPS *≥* 3	25.86	33.72	16.67	26.56	31.76		28.53
DRS *≥* 3, CPS < 3	16.17	20.00	19.76	25.68	29.51		19.52
DRS *≥* 3, CPS *≥* 3	34.69	28.91	33.33	28.59	27.25		28.54
*P* value	***	***	–	NS	NS		***
Total	16.24	24.25	19.24	26.64	29.6	***	21.82
Psychiatrist							
DRS < 3, CPS < 3	3.95	3.67	1.79	1.15	0		2.59
DRS < 3, CPS *≥* 3	10.34	4.99	0	2.41	4.71		3.44
DRS *≥* 3, CPS < 3	11.60	9.14	4.71	3.38	3.28		7.50
DRS *≥* 3, CPS *≥* 3	12.24	9.95	0	5.11	5.09		6.13
*P* value	–	***	–	–	–		***
Total	6.46	5.73	2.37	3.17	4.57	***	4.05
Day care center							
CPS < 3	0.20	1.28	1.80	5.80	3.26		1.53
CPS *≥* 3	0	2.62	0	8.27	10.10		7.32
*P* value	–	NS	–	NS	NS		***
Total	0.19	1.67	1.80	7.84	9.20	***	3.24

Table presents for each disability profile (*Low limit.* Low-level limitations, *IADL (cogn.)* IADL and low level of cognitive impairment, *Func* Functional limitations, *Func., cogn.* Functional & cognitive impairments, *func., cogn., behav.* Functional, cognitive & behavioural problems) the proportions of people utilizing the service. The p values in columns are derived from the chi-squared test comparing the disability profiles. The *p* values in lines are obtained from the chi-squared test comparing the different levels of DRS and/or CPS impairment. ***: *p* value ≤0.001; ** *p* value ≤0.01; *NS* No significant; −: minimum expected value < 5

Geriatricians were consulted more often by people with cognitive problems, whether these were combined with functional limitations or not. The more complex the disability profile, the more often a neurologist was consulted. For people with similar disability profiles, a neurologist was more frequently consulted by those with cognitive impairment. Neurologists were more frequently visited than geriatricians (13% versus 6% of the total sample) (Table 5). The association between visits to an emergency department or out-of-hours general practitioner visits (evening, weekend, or public holiday consultations) and the severity of disabilities was unclear. Beneficiaries of Protocol 3 with functional limitations were the biggest users of emergency departments (36.62% used an emergency department at least once a year), followed by beneficiaries with functional and cognitive impairment (32.96%), followed by beneficiaries with functional, cognitive, and behavioral problems (28.45%) and beneficiaries with IADL limitations and a low level of cognitive impairment (28.17%) (Table 5). Finally, up to 92.5% of people suffered from multi-morbidity (Table 5) and the number of morbidities was roughly similar in the different disability profiles, with a median number of comorbidities equal to 4 in all disability profiles.

**Table 5:** Global or acute IMA proxies by disability profile and level of ADL and CPS impairment

	Low limit.	IADL (cogn.)	Func.	Func., cogn.	Func., cogn., behav.	*P* value	Total
Geriatrician							
ADL < 3, CPS < 3	4.52	6.88	3.74	13.33	2.78		5.08
ADL < 3, CPS *≥* 3	13.33	13.56	0	13.65	16.88		14.05
ADL *≥* 3, CPS < 3	8.62	0	3.95	4.76	5.36		4.16
ADL *≥* 3, CPS *≥* 3	0	25	30	7.32	9.46		7.88
*P* value	–	–	–	–	–		***
Total	5.07	8.83	3.96	7.59	10.49	***	6.24
Neurologist							
ADL < 3, CPS < 3	8.01	11.36	11.23	6.67	2.78		9.81
ADL < 3, CPS *≥* 3	20.95	20.42	0	18.88	33.12		22
ADL *≥* 3, CPS < 3	12.07	0	9.71	12.28	16.07		10.18
ADL *≥* 3, CPS *≥* 3	0	0	10	17.71	23.42		18.77
*P* value	–	–	–	–	***		***
Total	8.76	13.93	10.15	16.86	23.99	***	12.89
General practitioner out-of-hours							
Total	15.84	20.85	26.78	26.33	19.4	***	23.08
Emergency department + hospitalization							
Total	22.06	28.17	36.62	32.96	28.45	***	31.04
Hospitalization							
Total	40.62	48.01	61.86	54.88	44.97	***	52.78
Multi-morbidity							
Total	91.24	91.01	93.63	93.67	90.66	***	92.53

Table presents for each disability profile (*Low limit.* Low-level limitations, *IADL (cogn.)* IADL and low level of cognitive impairment, *Func* Functional limitations, *Func., cogn.* Functional & cognitive impairments, *func., cogn., behav.* Functional, cognitive & behavioural problems) the proportions of people utilizing the service. The p values in columns are obtained from the chi-squared test comparing the disability profiles. The p values in lines are related to the chi-squared test comparing the different levels of ADL and CPS impairment. ***: *p* value ≤0.001; *NS* No significant; −: minimum expected value < 5

#### Disability profiles: adjusted comparison of healthcare utilization between groups studied

In spite of the extensive matching (Table 2), healthcare utilization related to disability profiles was significantly different between the intervention and control groups with respect to several types of healthcare services (Table 6). These differences were observed after adjustment for socioeconomic variables (Additional file 1 presents socioeconomic variables). Participants in the control group received significantly more personal nursing care and physical therapy and more of them received the incontinence lump sum. Parkinson’s medication was prescribed more often in the intervention group, except for individuals with functional, cognitive, and behavioral problems, for whom there was no difference. Neuroleptics, a specific medication for cognitive impairments, was used more in the intervention group than in the control group by those who did not have significant cognitive impairments. Individuals with low to severe levels of cognitive impairment used day care centers significantly more often in the intervention group than in the control group. Finally, people in the intervention group consulted more specialists and used the emergency department significantly more than those in the control group, regardless of their disability profile.

**Table 6:** Comparison of history of healthcare utilization (from IMA proxies) between groups studied

	Low limit.	IADL (cogn.)	Func.	Func., cogn.	Func., cogn., behav.
Functional IMA proxies					
Personal nursing cre	0.25 [0.22/0.29]	0.33 [0.28/0.38]	0.21 [0.18/0.23]	0.31 [0.26/0.37]	0.3 [0.23/0.39]
Physical therapy	0.88 [0.71/1.12]	0.77 [0.64/0.93]	0.6 [0.55/0.66]	0.83 [0.74/0.94]	0.48 [0.34/0.62]
Parkinson’s medication	3.76 [2.57/5.9]	1.78 [1.3/2.41]	1.31 [1.12/1.54]	1.47 [1.2/1.78]	1 [0.66/1.5]
Incontinence lump sum	0.15 [0.09/0.23]	0.15 [0.1/0.2]	0.24 [0.21/0.27]	0.32 [0.28/0.37]	0.16 [0.11/0.22]
Cognitive, mood IMA proxies					
Antidepressant	1.26 [1.1/1.44]	1 [0.88/1.17]	1.34 [1.22/1.49]	0.99 [0.88/1.11]	1.94 [1.52/2.47]
Neuroleptic	1.55 [1.26/1.9]	1.45 [1.21/1.75]	1.78 [1.53/2.06]	1.1 [0.97/1.26]	0.41 [0.31/0.53]
Neuropsychiatrist	3.56 [2.88/4.55]	2.82 [2.37/3.35]	3.19 [2.76/3.71]	4.89 [4.06/5.92]	28.69 [16.69/68.78]
Psychiatrist	0.83 [0.62/1.12]	4.5 [2.96/7.8]	2.93 [1.97/4.7]	1.68 [1.2/2.45]	3.07 [1.49/8.7]
Day care center	-[−/−]	6.31 [2.57/39.3]	1.22 [0.87/1.75]	2.17 [1.69/2.9]	2.18 [1.24/4.19]
Global IMA proxies					
Geriatrician	3.41 [2.42/5]	2.63 [2.02/3.49]	1.21 [0.95/1.52]	1.51 [1.19/1.93]	17.94 [8.54/81.03]
Neurologist	3.16 [2.47/4.21]	3.39 [2.64/4.47]	1.65 [1.38/1.99]	3.72 [2.98/4.65]	8.65 [5.54/17]
Hospitalization	1.39 [1.15/1.71]	2.46 [1.98/3.07]	2.21 [1.95/2.5]	2.24 [1.92/2.59]	4.13 [2.81/6.29]
Acute IMA proxies					
General prctitioner out-of-hours	3.44 [2.84/4.27]	2.64 [2.24/3.12]	3.98 [3.58/4.49]	2.86 [2.49/3.31]	3.11 [2.41/4.28]
Emergency department + hospitalization	2.92 [2.54/3.41]	1.85 [1.62/2.14]	2.77 [2.51/3.04]	3.01 [2.68/3.41]	2.43 [1.89/3.18]

The table reports for each disability profile (*Low limit.* Low-level limitations, *IADL (cogn.)* IADL and low level of cognitive impairment, *Func* Functional limitations, *Func., cogn.* Functional & cognitive impairments, *func., cogn., behav.* Functional, cognitive & behavioural problems) the odds ratios with their 95% confidence intervals adjusted for socio-demographic variables

## Discussion

**Fig. 1:**
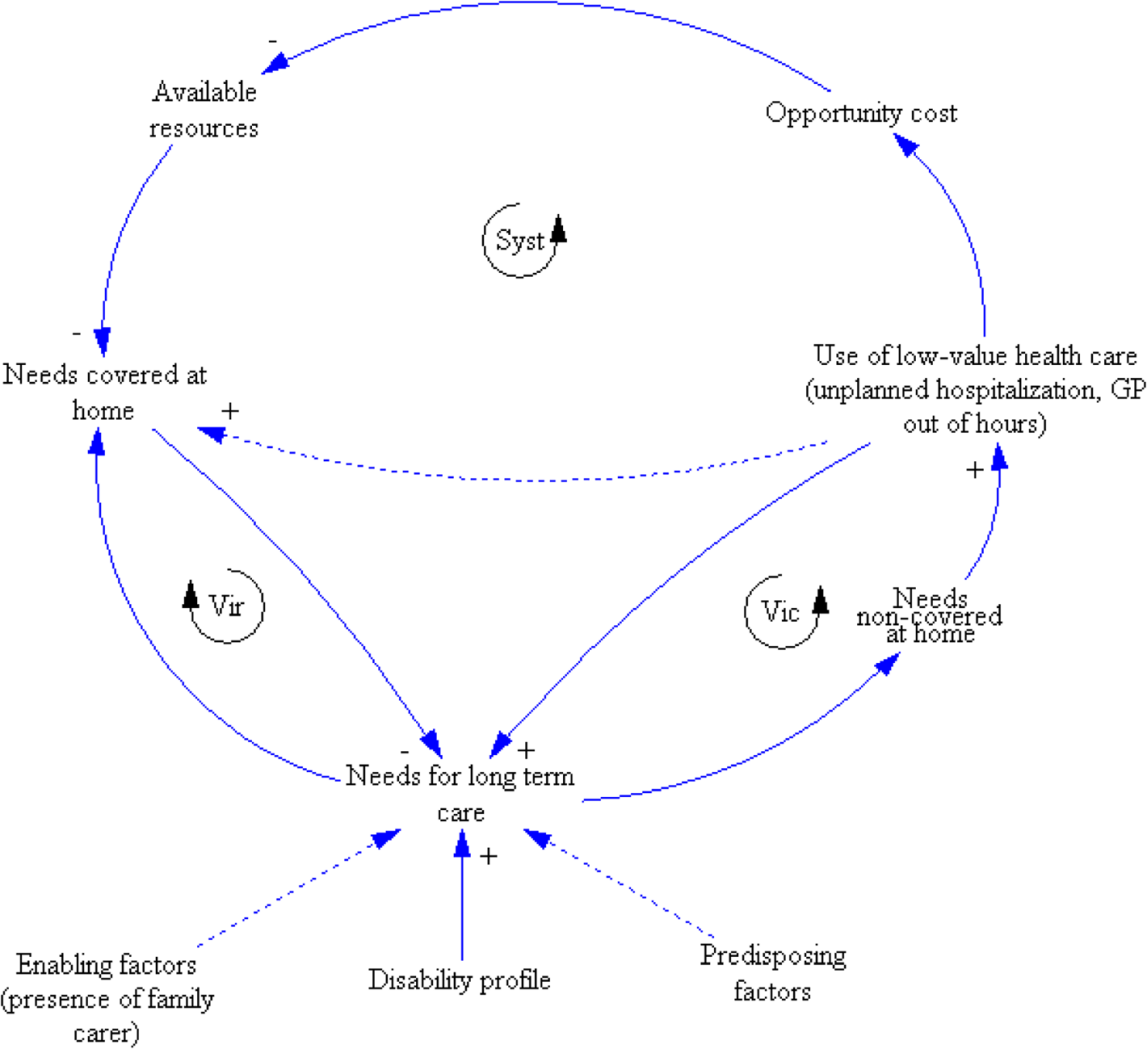
Individual and systemic healthcare utilization patterns determined by combining disability profiles and healthcare utilization

As argued in the introduction, schematized in Fig. 1, and described in Anderson’s socio-behavioral model of health service utilization [47], the need for and the utilization of long-term care depend on predisposing factors associated with the likelihood of needing health services (e.g.. age or gender), on enabling factors (including the availability of health facilities and the capacity to avail of health services), and on health-related factors such as the level of disability [48]. In the context of aging, a crucial enabling factor for utilization of long-term care is social support and especially the living arrangements of a family carer. The living arrangements of a family carer influence both informal and formal care [26], as a co-resident family carer is more involved in help (i.e. intimate care) [49], while a non-resident family carer is more likely to call on health and social care services [39].

As the results showed that older persons with similar disability profiles don’t necessary utilize healthcare in similar ways, the coverage of long-term care needs could be described by two distinct pathways when healthcare utilization and disability profiles are combined. First, a virtuous pathway (Vir loop) is present when the needs for health and social care are covered. The coverage of these needs diminishes future needs and lessens the use of services that have low value. For instance, it is known that the use of both a primary physician and primary care services along with a high level of continuity of care are associated with lower rates of emergency department use [50]. Second, a vicious loop is initiated by a lack of coverage of long-term care needs, leading to an overuse of low-value services (unplanned hospitalization, out-of-hours general practitioner visits); this results either in an increase in needs (Vic loop) or a break of the vicious loop when a response is provided to initial needs (dotted arrow). This vicious loop is well documented for emergency department use. People with unmet healthcare needs and without primary and supportive care services have been shown to visit emergency departments more often. These people are extremely vulnerable and the focus on acute problems in emergency departments and inadequate follow-up can lead to recurring emergency department visits, subsequent hospitalizations, institutionalization, or even death [51].

The potential gain obtained from the coverage of previously uncovered needs by Protocol 3 interventions is not limited to the individual; these interventions can also substantially reduce the pressure on the health system. This has been supported by literature suggesting that a significant proportion of emergency department visits by older people could potentially be prevented by better primary healthcare [52]. It has also been shown that older people take up a higher proportion of emergency department use and of hospital stays [51]. Their emergency department visits are more complex and more likely to result in hospitalization than those of younger people [53].

The consequences of P3 interventions on the individual vicious loop and the systemic vicious loop can be studied adequately, provided that the three essential questions in conducting comparative effectiveness studies, and at the basis of this paper’s objective, can be tackled.

We will now review key lessons and elements of responses to these questions in the light of our results.

### Is it possible to identify the need for long-term care? Can this lead to a meaningful stratification of frail older people and a clear definition of a control group?

The stratification of the study population is often presented in the literature as a crucial process in order to test the effectiveness of interventions and their adaptation to the specific needs of people within groups [54]. The five disability profiles created in this study identify groups with specific long-term care needs [30].

A control group with similar characteristics in term of disability and predisposing and enabling factors was established. However, the groups studied differ in terms of healthcare utilization. The study sheds light on the difficulties involved in combining disability profiles and healthcare utilization to define the control group, in the actual context of available Belgian data.

### To what extent does the utilization of long-term care match the need for long-term care?

Our results show that a database of healthcare reimbursements is not a good proxy for disability profiles (which make possible the identification of needs for long-term care). Not only are these data not specific enough to differentiate between disability profiles, but people with similar disability profiles do not necessarily utilize healthcare in similar ways.

A partial explanation of this difference could be the occurrence of acute health problems versus a progressive deterioration of health status. In our case, hospitalization within the 2 months before inclusion in the cohort has been used as a proxy for deterioration of health. This deterioration could mainly result either from acute problems for which the patient was being hospitalized [55] or from “iatrogenic disability”, defined as “the avoidable dependency which often occurs during the course of care” [56]. The disability profile of older people without recent hospitalization was considered to be the consequence of a progressive deterioration and not the result of a recent acute problem. For people with such a profile, differences between the intervention and control groups could be viewed as underutilization of the healthcare system or as mismanagement of people’s health problems.

Another explanation could be the social characteristics. Anderson’s socio-behavioral model of health service utilization proposes a series of predisposing factors alongside age and gender as social structure components, the health beliefs and a series of other enabling factors alongside the living arrangements of family career as the community ressources, the organization of health and social care, the knowledge of the services, the autonomy in their utilization, the income, the supplementary health insurance,... These factors should be integrated to the analysis to better understand the utilization and the need for health services [47]. However, these variables are not available.

To obtain more accurate information on disability profiles and more generally on health status, Electronic Health Records (EHR) may be of use in the future. However, in Belgium, these are not yet used by every practitioner and therefore they are not routinely available for scientific research [57]. The whole system is actually in development (EHR and pooling of information for population health and scientific studies), in Belgium. It is nevertheless not expected to include information collected by social worker. Indeed, in Belgium, due to a split decision-making power between the federal authorities and the federated entities [46, 58] the organization and the funding of formal social services are separated from health care services. These information are even though essential to better support frail older persons at home.

### Is it possible to use routine databases of healthcare reimbursement to select controls with long-term care needs similar to those of the intervention groups?

If the objective is to assess the effectiveness of an intervention, the easiest way to evaluate it is to compare the intervention group to a control group with similar baseline characteristics (in our case, with similar long-term care needs) [59]. As discussed earlier, the baseline characteristics must include disability profiles and healthcare utilization. Indeed, a similar disability profile allows us to evaluate the impact of the intervention on the evolution of clinical outcomes and on long-term care needs. On the other hand, similar baseline healthcare utilization facilitates the evaluation of the consequences of the interventions for how widely long-term care needs are met and for reducing the use of low-value healthcare services. Such a reduction can be interpreted as a decrease of the pressure on the healthcare system.

As shown in this study, some proxies for healthcare utilization appeared to be associated with some disability profiles. However, this association was not clear enough to allow the determination of disability profiles from healthcare utilization. So, in our study, we were not limited by the “technical” capacity to use variables in the control group (indeed, the propensity score method does not limit the number of control variables [60]). The limitations of this study are related to the availability of individually measured variables in the control group.

There were two difficulties related to the scarcity of the variables required to create an “ideal” control group. First, people in the intervention group were recruited through different entry points, including at discharge from hospital, from the waiting lists of the nursing homes, and from other sources. People in the control group were recruited from among those receiving nursing care at home, for organizational reasons. The different recruitment points constituted an important potential for a selection bias. However, they make it possible to appreciate the importance of integrating healthcare utilization into the discussion about unmet long-term care needs. Second, the number of controls was considerably smaller than the number in the intervention group, due to resource constraints. The method of one-by-one matching of the nearest neighbors with replacement that was used in this study accounted for the replication of certain controls. Other statistical approaches were considered, including bootstrapping on the matching or changing the ratio of Protocol 3beneficiaries to controls. However, these alternatives did not entirely resolve the scarcity of some profiles within the control group. The best solution would have been to include more controls from diverse entry points, which would have demanded considerable time and financial resources. Another option would be to exclusively use more diverse routine data (such data is either not yet available in Belgium or else links cannot yet be made between such data) to establish the control group. Indeed, routine data are recognized as the best tool for evaluating large-scale interventions. They are recommended in comparative effectiveness studies [61], as they are available for the whole population. The use of routine data also makes it possible to study real-world effectiveness [62, 63] and helps in policy- and decision-making, both through prediction of future needs and comparison with other public programs [64].

In Belgium, routine data provides information on reimbursed healthcare consumption, including healthcare utilization and proxies for comorbidities, but not disability profiles. These data can therefore help track the utilization of healthcare services over time, but they give no idea of long-term care needs and the coverage of those needs. Nevertheless, Belgium is trying to reorganize its health system and hopes to draw on the expertise gained from Protocol 3 projects to take a step forward in the implementation of projects designed to provide integrated care for people with chronic health problems [58]. These integrated care projects should involve a comprehensive reorganization of healthcare and social services, as well as the pooling of resources and policy adjustments. Such projects can only be successful if, at the same time, appropriate tools for data sharing are developed. These tools may be used for assessing the needs of patients and for evaluating healthcare utilization (by using population data). Clinical scales may help to better characterize disability profiles.

## Conclusions

This paper raised three key questions that need to be addressed in order to understand the relationship between individual disability status, patterns of use of healthcare services, and the effectiveness of interventions. To explore the first question, we discussed an approach to stratifying and establishing a control group for the assessment of the effectiveness of interventions targeting frail older people at home. As a consequence, and in response to the second question, this study sheds more light on the association between disability profiles and healthcare utilization. Finally, and in relation to our third question, this study encourages both researchers and policy-makers to reflect on the best trade-off between using large databases with a limited number of available variables (such as healthcare utilization data without disability variables) and a relatively small sample with a sufficient number of variables (disability variables) specifically collected in an observational comparative effectiveness study. These results therefore represent an important contribution to future designs of comparative effectiveness studies. This contribution is all the more relevant at a time when important transformations in electronic records on health and healthcare use and resulting databases are under scrutiny in Belgium and other European countries. Our findings should definitely pave the way for better use of new routine databases to evaluate interventions.

## Additional file

Additional file 1: Socio-demographic characteristics of the groups studies by disability profile. (DOCX 25 kb)

## Data Availability

The datasets generated and/or analyzed during the current study are not publicly available due to recommendations made by the Belgian privacy commission. However, any person can apply for access to the data through the Belgian privacy commission.

## Abbreviations

ABS: Aggressive Behavior Scale
ADL: Activities of daily life
CPS: Cognitive performance scale
DRS: Depression rating scale
EHR: Electronic Health Records
FC: Family carer
Func: Functional
Func., cogn.: Functional & cognitive
Func., cogn., behav.: Functional, cognitive & behavioural problems
GMVR: Geometric Mean Variance Ratio
GP: General practitioners
IADL (cogn.): IADL and low level of cognitive impairment
IADL: Instrumental activities of daily life
IMA: Inter-mutuality Agency
Low limit.: Low-level limitations
NIHDI: National Insurance for Health and Disability Institute
PCA: Principal Component Analysis
SMD: Average Standardised Mean Difference
smd: Standardised mean difference
Vic loop: Vicious loop
Vir loop: Virtuous loop
VR: Variance ratio
WHO: World Health Organization
