# Supplementary material for: Evaluation of bottom-up interventions targeting community-dwelling frail older people in Belgium: methodological challenges and lessons for future comparative effectiveness studies
Source: BMC Health Serv Res. 2019 Jun 24;19:416. doi: 10.1186/s12913-019-4240-9 (PMC6592000; doi:10.1186/s12913-019-4240-9)
Supplement: Supplementary file 1 — Socio-demographic characteristics of the groups studies by disability profile. (DOCX 25 kb) [file 12913_2019_4240_MOESM1_ESM.docx]

**Additional file 1.** Socio-demographic characteristics of the groups studies by disability profile

|  | Low limit. | | IADL (cogn.) | | Func. | | Func., cogn. | | Func., cogn., behav. | | Total | |
| --- | --- | --- | --- | --- | --- | --- | --- | --- | --- | --- | --- | --- |
|  | Treated | Control | Treated | Control | Treated | Control | Treated | Control | Treated | Control | Treated | Control |
| **Age** | | | | | | | | | | | | |
| Median [IQR] | 80[73-84] | 83[73-88] | 81[76-86] | 84[77-88] | 82[76-86] | 83[78-88] | 82[77-87] | 83[76-88] | 80[73-85] | 82[75-88] | 81[76-86] | 83[77-88] |
| P value |  | *** |  | *** |  | *** |  | * |  | *** |  | *** |
| **Gender** |  |  |  |  |  |  |  |  |  |  |  |  |
| % Men | 30.39 | 20.2 | 28.42 | 33.23 | 27.35 | 28.74 | 38.43 | 34.2 | 47.92 | 42.58 | 31.78 | 29.96 |
| % Women | 69.61 | 79.8 | 71.58 | 66.77 | 72.65 | 71.26 | 61.57 | 65.8 | 52.08 | 57.42 | 68.22 | 70.04 |
| P value |  | *** |  | ** |  | NS |  | ** |  | NS |  | ** |
| **Family carer** | | | | | | | | | | | | |
| % Without | 36.27 | 36.27 | 13.82 | 13.82 | 15.07 | 15.07 | 5.22 | 5.22 | 4.3 | 4.3 | 16.07 | 16.07 |
| % non-resident. | 47.3 | 47.3 | 58.75 | 58.75 | 58.47 | 58.47 | 39.68 | 39.68 | 31.75 | 31.75 | 50.7 | 50.7 |
| % co-resident | 16.42 | 16.42 | 27.43 | 27.43 | 26.46 | 26.46 | 55.09 | 55.09 | 63.95 | 63.95 | 33.23 | 33.23 |
| P value |  | NS |  | NS |  | NS |  | NS |  | NS |  | NS |
| **Region** | | | | | | | | | | | | |
| Brussels | 11.13 | 0.44 | 6.88 | 0 | 2.2 | 0.26 | 2.85 | 0 | 4.6 | 0 | 5.02 | 0.18 |
| Flanders | 61.18 | 65.1 | 65.53 | 40.37 | 84.06 | 77.41 | 77.25 | 75.65 | 72.7 | 61.13 | 74.24 | 67.05 |
| Wallonia | 27.7 | 34.46 | 27.59 | 59.63 | 13.74 | 22.32 | 19.91 | 24.35 | 22.7 | 38.87 | 20.75 | 32.77 |
| P value |  | *** |  | *** |  | *** |  | *** |  | *** |  | *** |
| **Median income** | | | | | | | | | | | | |
| Low | 22.89 | 19.36 | 18.32 | 31.42 | 10.49 | 18.42 | 11.66 | 11.66 | 13.35 | 26.11 | 14.67 | 19.96 |
| Medium | 61.03 | 57.79 | 62.53 | 41.36 | 66.81 | 56.08 | 56.43 | 57.3 | 55.04 | 54.6 | 61.99 | 53.94 |
| High | 16.08 | 22.84 | 19.15 | 27.23 | 22.69 | 25.49 | 31.91 | 31.04 | 31.6 | 19.29 | 23.34 | 26.11 |
| P value |  | *** |  | *** |  | *** |  | NS |  | *** |  | *** |
| **N** | | | | | | | | | | | | |
| N | 2040 | 120 | 1932 | 149 | 3821 | 395 | 2316 | 207 | 674 | 35 | 10783 | 599 |

Legend: Table presents socio-demographic characteristics of the groups studied (treated and control groups) by disability profile (Low limit.: low-level limitations; IADL (cogn.): IADL and low level of cognitive impairment; Func: functional limitations; Func., cogn.: functional & cognitive impairments; Func., cogn., behav.: functional, cognitive & behavioural problems). Median [IQR]: median with its interquartile range; N: number of participants. The other numbers are the proportions of individuals in the different socio-demographic categories. The p value is obtained from the chi-squared test comparing different socio-demographic categories. ***: p value ≤ 0.001, **p value ≤ 0.01; *p value ≤ 0.05; NS: No Significant; - : minimum expected value < 5.

For the most part, the control group was relatively similar to the intervention group. Study participants were significantly older in the control group than in the intervention group. Gender distribution was slightly unbalanced for the "low limitation", "IADL and initial cognitive impairment", and "functional and cognitive impairment" disability profiles. The majority (62%) of people in the intervention group lived in municipalities with a medium median income and 15% lived in communes with a low median income. In the control group, the proportions of individuals living in municipalities with low, medium, and high median income were more representative of the population of Belgium, with 54% of study participants in this group living in municipalities with a medium median income, 20% in municipalities with a low median income, and 26% in municipalities with a high median income.
